# Supplementary material for: Sensitive inference of alignment-safe intervals from biodiverse protein sequence clusters using EMERALD
Source: Genome Biol. 2023 Jul 17;24:168. doi: 10.1186/s13059-023-03008-6 (PMC10351170; doi:10.1186/s13059-023-03008-6)
Supplement: Supplementary file 1 — Additional file 1: Figure S1. Safety coverage restricted on each structural type as assigned by STRIDE (i.e., for every structural type A (number of amino acids of type A that are safe) / (total number of amino acids of type A)). [file 13059_2023_3008_MOESM1_ESM.pdf]

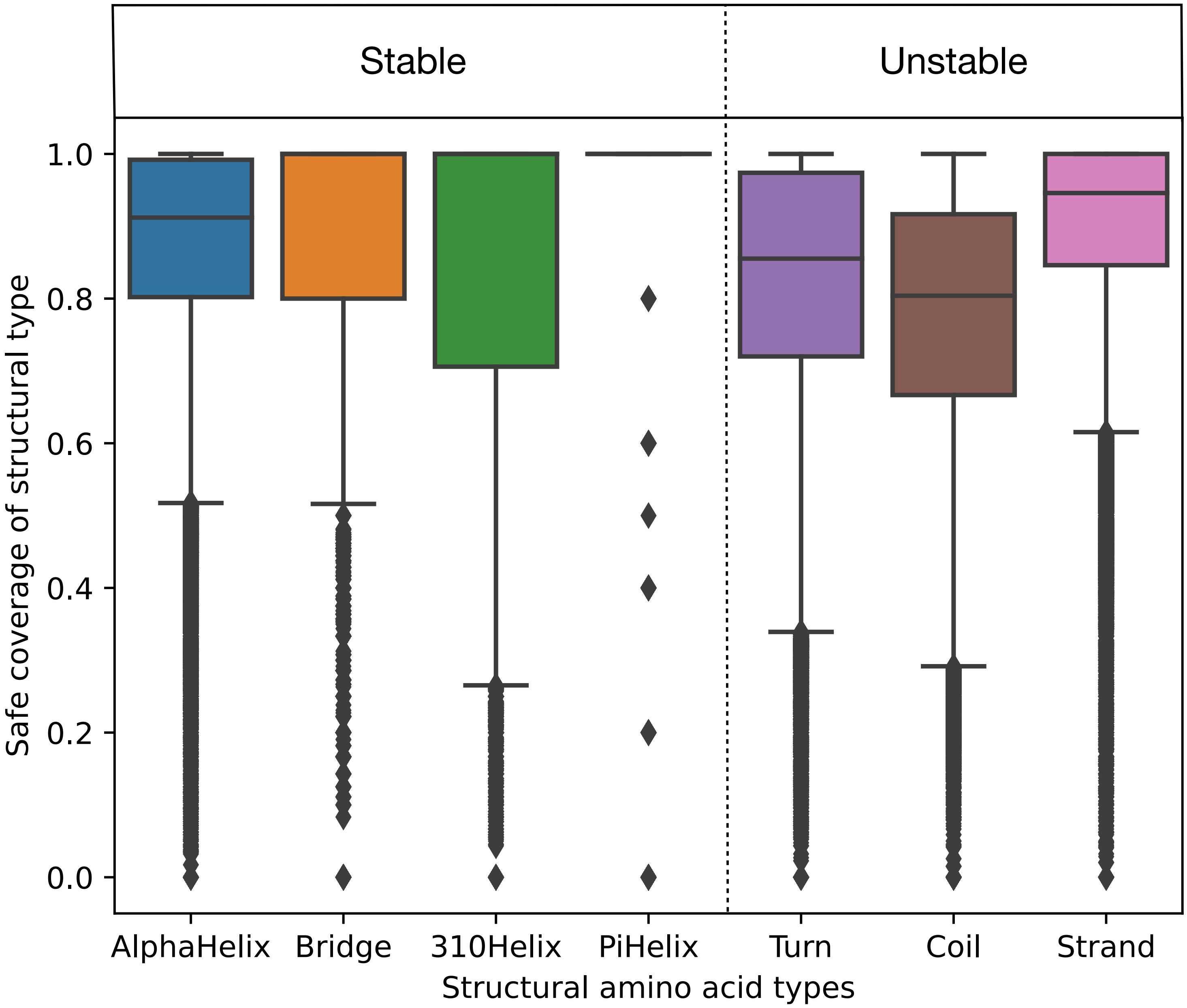


Figure S1: Safety coverage restricted on each structural type as assigned by STRIDE (i.e. for every structural type $A$ (number of amino acids of type $A$ that are safe) / (total number of amino acids of type $A$)) with EMERALD parameters $\alpha = 0.75$ and $\Delta = 8$, throughout all 400k sequences, including previously removed 4k sequences which did not include any stable amino acids. Every point is the safety coverage restricted to the corresponding structure type for all safety windows of a sequence. In the experimental results, we defined Turn, Coil and Strand as unstable, and all other structural types as stable. For example, AlphaHelix has a median safety coverage of 90% throughout all sequences. We observe that stable amino acids have a larger safety coverage than unstable ones, with the exception of Strand having a higher coverage than AlphaHelix. The distribution of the structural types throughout all sequences is as follows, rounded to one decimal place: AlphaHelix: 40.3%, Bridge: 0.8%, 310Helix: 3.2%, PiHelix: 0.007%, Turn: 17.7%, Coil: 19.1% and Strand: 18.7%.
