# Supplementary material for: Sensitive inference of alignment-safe intervals from biodiverse protein sequence clusters using EMERALD
Source: Genome Biol. 2023 Jul 17;24:168. doi: 10.1186/s13059-023-03008-6 (PMC10351170; doi:10.1186/s13059-023-03008-6)
Supplement: Supplementary file 2 — Additional file 2: Figure S2. Stable structure retention compared to safety coverage for various EMERALD parameter configurations and several identity ranges. [file 13059_2023_3008_MOESM2_ESM.pdf]

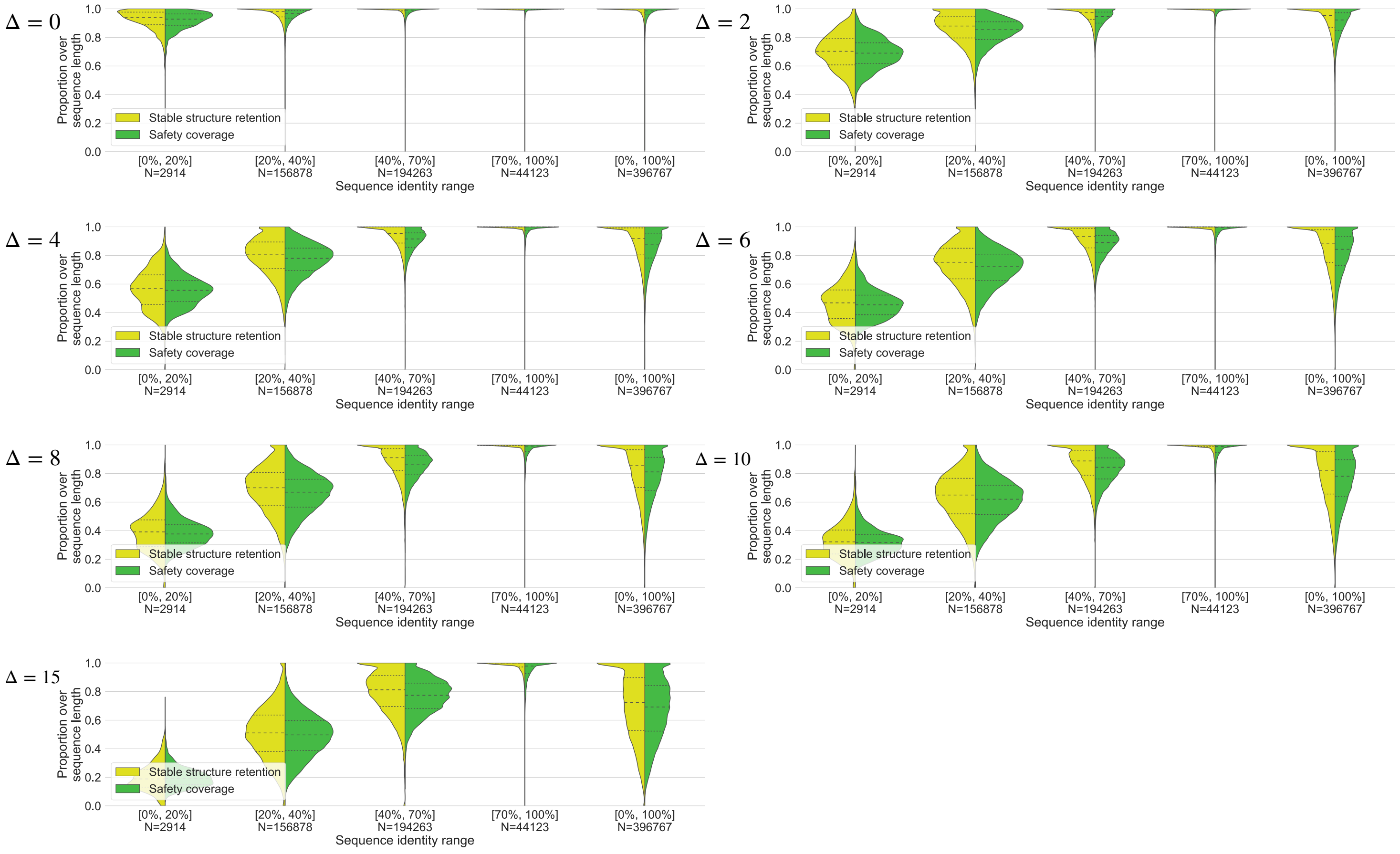

Figure S2: Stable structure retention compared to safety coverage for $\alpha = 1$, $\Delta = 0, 2, 4, 6, 8, 10, 15$ and several identity ranges. The median and the quartiles are indicated in black.
