# Supplementary material for: Sensitive inference of alignment-safe intervals from biodiverse protein sequence clusters using EMERALD
Source: Genome Biol. 2023 Jul 17;24:168. doi: 10.1186/s13059-023-03008-6 (PMC10351170; doi:10.1186/s13059-023-03008-6)
Supplement: Supplementary file 5 — Additional file 5: Table S1. Summary statistics of pairwise sequence assessments in regard to their parameter-specific safety-window length construction. [file 13059_2023_3008_MOESM5_ESM.pdf]

Parameters	ID range	Number of sequences	Number of safety windows	Average length of sequences	Average length of safety windows	Proportion of safety windows of length $\geq 50\%$
$\alpha = 0.51, \Delta = 8$	$[0\%, 100\%]$	400408	2882661	321.89	40.49	6.9%
$\alpha = 0.75, \Delta = 0$	$[0\%, 100\%]$	400408	1059216	321.89	117.96	30.5%
	$[0\%, 20\%]$	2922	16674	467.74	75.58	5.3%
	$[20\%, 40\%]$	157236	551251	339.47	92.50	18.9%
	$[40\%, 70\%]$	197051	433730	308.90	137.17	40.4%
	$[70\%, 100\%]$	44674	61172	304.80	221.11	71.7%
$\alpha = 0.75, \Delta = 2$	$[0\%, 100\%]$	400408	2075297	321.89	56.67	10.5%
$\alpha = 0.75, \Delta = 4$	$[0\%, 100\%]$	400408	2375169	321.89	47.87	8.3%
$\alpha = 0.75, \Delta = 6$	$[0\%, 100\%]$	400408	2532554	321.89	43.66	7.4%
$\alpha = 0.75, \Delta = 8$	$[0\%, 100\%]$	400408	2643650	321.89	40.73	6.8%
	$[0\%, 20\%]$	2922	52110	467.74	14.70	0.03%
	$[20\%, 40\%]$	157236	1522093	339.47	26.27	1.7%
	$[40\%, 70\%]$	197051	989107	308.90	54.51	11.3%
	$[70\%, 100\%]$	44674	88974	304.80	149.21	47.5%
$\alpha = 0.75, \Delta = 10$	$[0\%, 100\%]$	400408	2784429	321.89	37.67	6.1%
$\alpha = 1.00, \Delta = 8$	$[0\%, 100\%]$	400408	1844829	321.89	53.41	9.4%

Table S1: Summary statistics of pairwise sequence assessments in regard to their parameter-specific safety-window length construction. Shown are the arithmetic means of aligned sequence lengths and their respective safety window counts and average safety-window lengths for $\sim$400k SwissProt sequences. In this assessment, we also included the previously removed $\sim$4k sequences which did not include any stable bases. We observe that when increasing the Δ parameter, this leads to increases in the number of safety windows while decreasing their average length. On the other hand, when increasing α, we observe a decrease in the number of safety windows and an average increase of safety-window lengths.
