# Supplementary material for: Sensitive inference of alignment-safe intervals from biodiverse protein sequence clusters using EMERALD
Source: Genome Biol. 2023 Jul 17;24:168. doi: 10.1186/s13059-023-03008-6 (PMC10351170; doi:10.1186/s13059-023-03008-6)
Supplement: Supplementary file 6 — Additional file 6: Figure S5. Stable structure overlap for each pair of α = 0.51, 0.75, 1 and Δ = 0, 2, 4, 6, 8, 10, 15 on the full dataset covering all 396k SwissProt sequences. [file 13059_2023_3008_MOESM6_ESM.pdf]

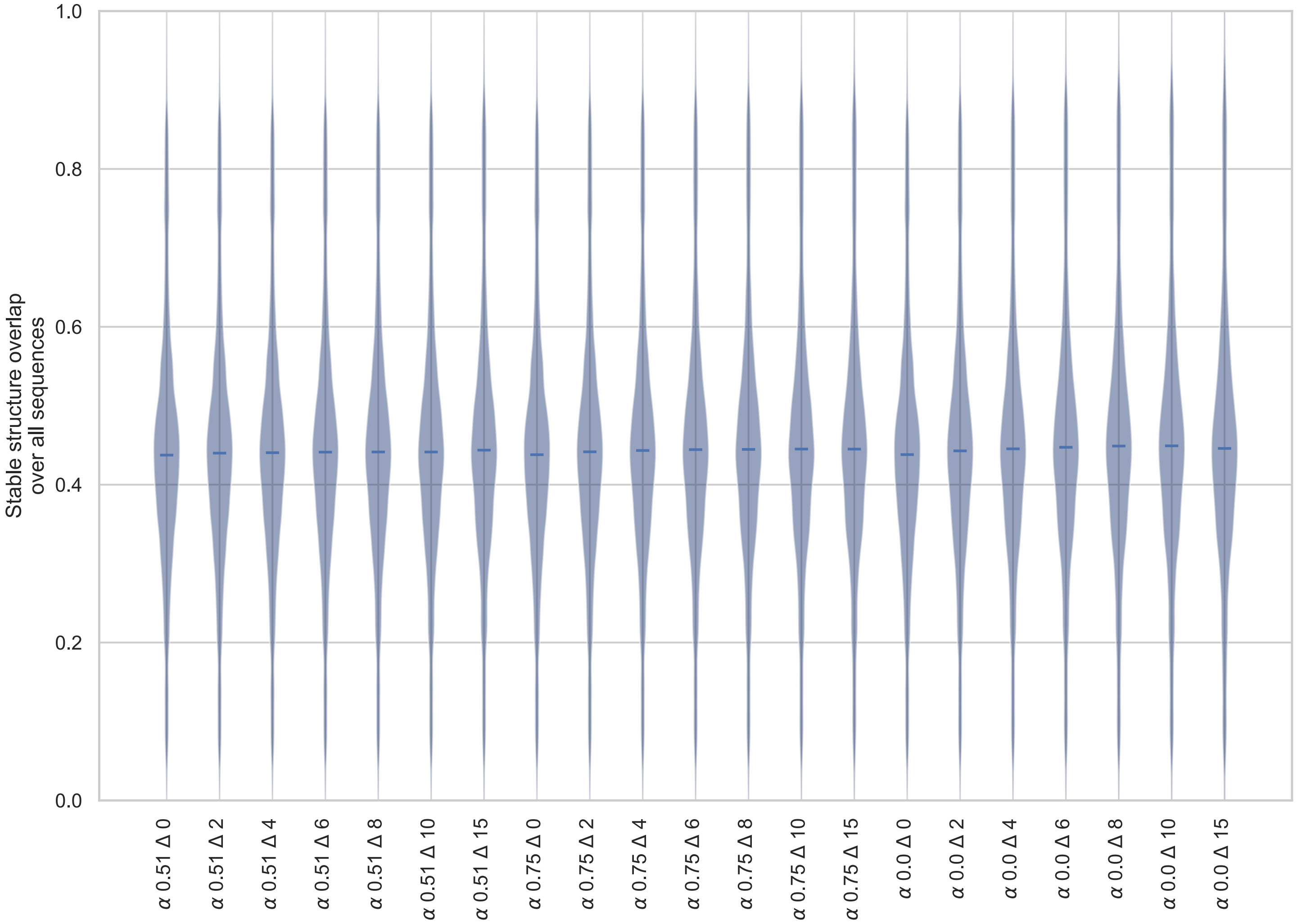

Figure S5: Stable structure overlap for each pair of $\alpha = 0.51, 0.75, 1$ and $\Delta = 0, 2, 4, 6, 8, 10, 15$ on the whole dataset of 396k sequences. 16 pairs of aligned sequences had to be filtered out due to a safety coverage of 0. The stable structure overlap is throughout all pairs constant with the median at 43%. This is exactly the proportion of stable positions within α-safety.
