# Supplementary material for: Sensitive inference of alignment-safe intervals from biodiverse protein sequence clusters using EMERALD
Source: Genome Biol. 2023 Jul 17;24:168. doi: 10.1186/s13059-023-03008-6 (PMC10351170; doi:10.1186/s13059-023-03008-6)
Supplement: Supplementary file 7 — Additional file 7: Figure S6. F1-score for α = 0.75, Δ = 8, for sequences in clusters of varying identity ranges. [file 13059_2023_3008_MOESM7_ESM.pdf]

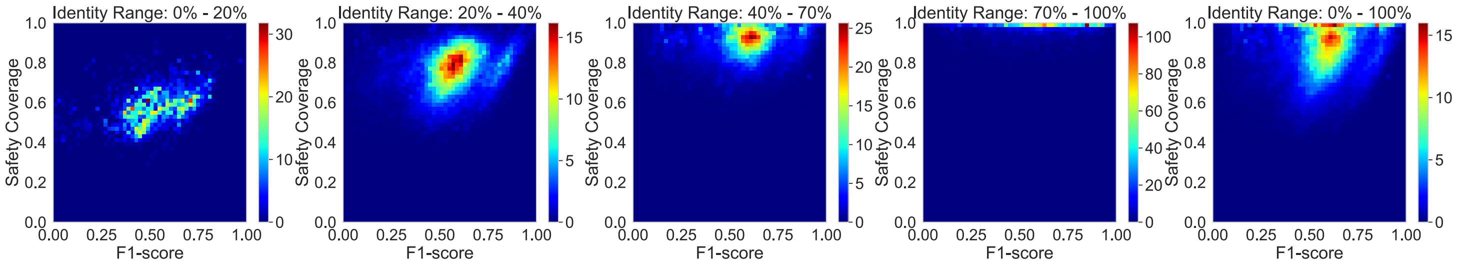

Figure S6: F1-score for $\alpha = 0.75$, $\Delta = 8$, for sequences in clusters of varying identity ranges. We can see that the F1-score lies consistently in a range around 50%, while in the lower cluster identities the sequence is covered less by safety windows. This indicates that EMERALD performs better in the identity range 20-70%.
