# Supplementary material for: Sensitive inference of alignment-safe intervals from biodiverse protein sequence clusters using EMERALD
Source: Genome Biol. 2023 Jul 17;24:168. doi: 10.1186/s13059-023-03008-6 (PMC10351170; doi:10.1186/s13059-023-03008-6)
Supplement: Supplementary file 8 — Additional file 8: Figure S7. Direct comparison of sequence logos between multiple sequence alignments (MSAs) and one-vs-all (cluster members against cluster representative) EMERALD alignments derived from one DIAMOND DeepClust clusters with various identity ranges (90% identity, 40% identity, 20% identity). [file 13059_2023_3008_MOESM8_ESM.pdf]

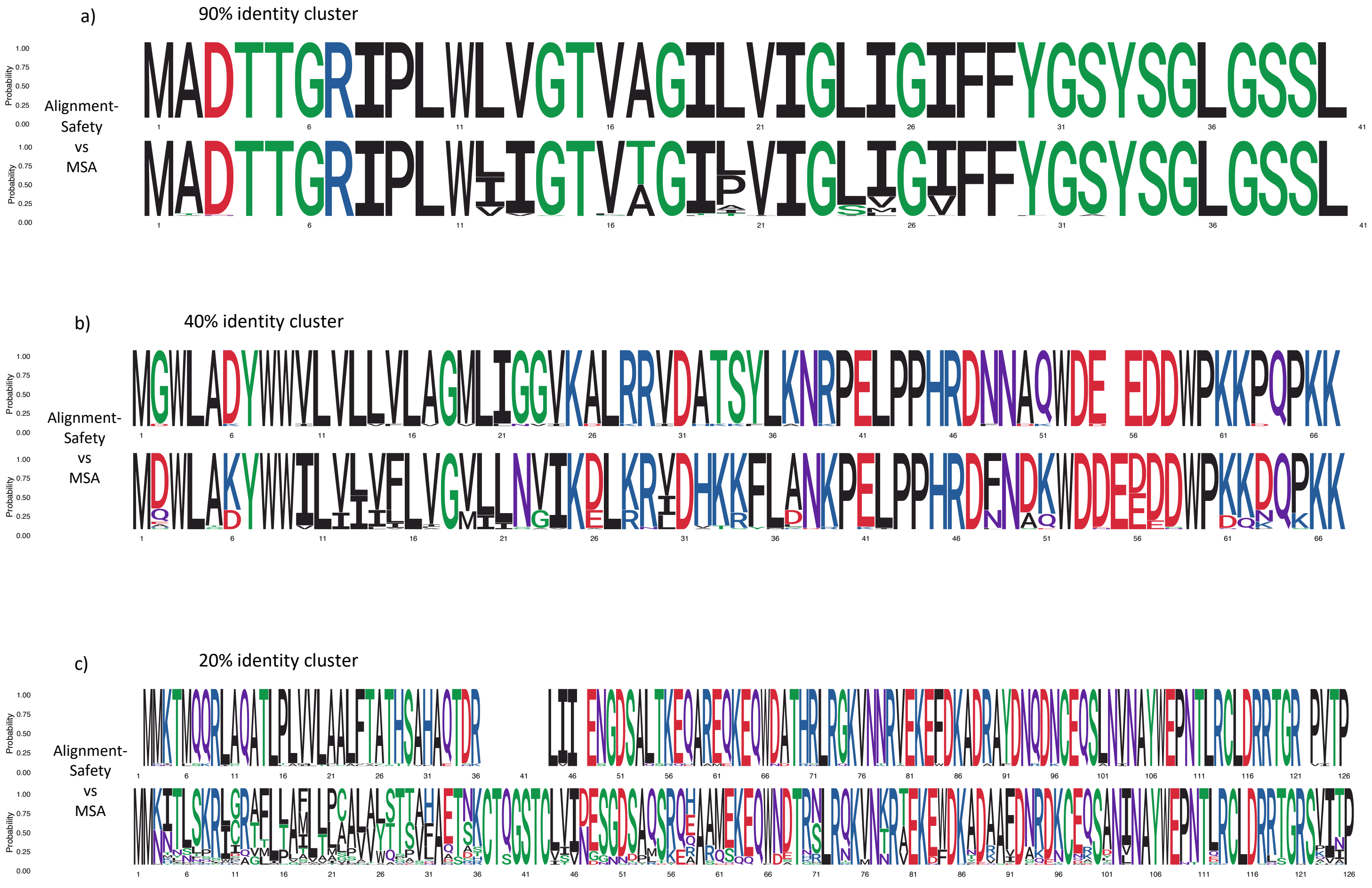

Figure S7: Direct comparison of sequence logos between multiple sequence alignments (MSAs) and one-vs-all (cluster members against cluster representative) EMERALD alignments derived from one DIAMOND DeepClust clusters with various identity ranges (90% identity, 40% identity, 20% identity). For MSAs, the sequence logos are generated directly from the MSA output, while for EMERALD alignments sequence logos are generated via interpolation of the conserved safety windows among different pairwise alignments (cluster representative vs cluster members). In the x-axis, the position of each amino acid is represented, while the y-axis has the probabilities defined by the relative frequencies of residue occurrence. Results are shown for a) (90% cluster identity), b) (40% cluster identity) and c) (20% cluster identity), the top sequence logos denote EMERALD derived alignments and the bottom sequence logo are generated from MSAs calculated using MUSCLE. Lower identity clusters contain larger proportions of biodiverse protein sequences. With increasing sequence diversity and lower identity boundaries in a cluster, the multiple sequence aligner attempts to optimally place alignment configurations such that the alignment of common regions is favoured. In contrast, sequence logos created with EMERALD show only the common regions that are intrinsically present throughout all pairwise alignments (or most alignments for $\alpha < 1$) and by the definition of safety, it is guaranteed that such a conserved region is present in at least α suboptimal alignments. Overall, this figure illustrates that conserved regions obtained from MSAs and EMERALD increasingly differ with decreasing identity boundaries, thereby presenting opportunities for subsequent studies to explore the differences in biological interpretations when comparing information derived from the suboptimal alignment space (alignment-safety) and information derived from MSAs.
