# Supplementary material for: Sensitive inference of alignment-safe intervals from biodiverse protein sequence clusters using EMERALD
Source: Genome Biol. 2023 Jul 17;24:168. doi: 10.1186/s13059-023-03008-6 (PMC10351170; doi:10.1186/s13059-023-03008-6)
Supplement: Supplementary file 9 — Additional file 9: Algorithm 1. Pseudocode denoting the algorithmic procedure underlying EMERALD for inferring alignment-safety windows from pairwise protein sequence alignments. [file 13059_2023_3008_MOESM9_ESM.pdf]

Algorithm 1: Pseudocode for inferring alignment-safety windows

Input : Two strings A and B, $\alpha \in (0.5, 1]$

Output: All α-safety windows of A and B, as subpath intervals in a path as in Lemmas 1 and 2 in $G(A, B)$

// We define $p(e)$, $p(P)$ as in eq. (2,3)

Construct $G_0(A, B) = (V_0, E_0)$;
for $v \in V_0$ **do**
    Compute $d(v)$ and $d_r(v)$; // As in eq. (1)
for $e \in E_0$ **do**
    Mark e as α-safe if $p(e) \geq \alpha$;
Find one s-t path $P^* = (v_0, v_1, \ldots, v_{k-1})$ in $G_0(A, B)$ containing all α-safe edges; // As in Lemma 2
$W \leftarrow \emptyset$;
$L \leftarrow 0, R \leftarrow 0$;
// Find maximal safe paths in P^*
while $R < k$ **do**
    while $L < R$ **and** $p(P^*[v_L..v_R]) < \alpha$ **do**
        $L \leftarrow L + 1$;
    if $L < R$ **and** $p(P^*[v_L..v_{R+1}]) < \alpha$ **then**
        $W \leftarrow W \cup \{[L, R]\}$;
    $R \leftarrow R + 1$;
return P^* and W.
