# Supplementary material for: Sensitive inference of alignment-safe intervals from biodiverse protein sequence clusters using EMERALD
Source: Genome Biol. 2023 Jul 17;24:168. doi: 10.1186/s13059-023-03008-6 (PMC10351170; doi:10.1186/s13059-023-03008-6)
Supplement: Supplementary file 10 — Additional file 10. Review history. [file 13059_2023_3008_MOESM10_ESM.docx]

Review History

**First round of review**

**Reviewer 1**

**Were you able to assess all statistics in the manuscript, including the appropriateness of statistical tests used?**

**Yes:** One metric isn't

**Were you able to directly test the methods?**

No

**Comments to author:**

Overall, I think that the motivation for the package is scientifically sound and interesting. I have a specific project in mind which would benefit from using this package.

Major changes:
- One thing that would be of interest and would demonstrate whether this method is valuable is the distribution of the lengths of the contiguous stable intervals. If the method ends up picking lots of very short robust matches it may be less useful than having longer contiguous intervals. I'm sure this is going to change a lot on the basis of the parameter choice too, so it would be good to help the reader see how to pick these parameters for various applications.
- While the code is available for the pipeline with DIAMOND, etc, as far as I can see, the benchmarking code isn't available/re-usable.
- Since you are doing it anyways, why not report the proportion of stable sequence by stride assignment (EMERALD-safe bases in AlphaHelix/Total AlphaHelix bases, etc). Boom, now you have a really interesting new scientific discovery with you tool to showcase an application of your method. This is the obvious scientific question raised by your method and it would save people a lot of effort/time to just report these results, since this is already calculated!
- You clearly compare against the obvious control (delta=1). However, I think it may also be important to compare against a multiple sequence alignment, as comparing multiple sequences at the same time may help "bridge the gap" between poorly aligned/divergent sequences.


Easy fixes, but extremely important changes:
- You need to include x- and y- axis labels on all figures! It is very annoying to have to read the legend to figure this out.
- You have to capitalise ALL your deltas. I thought it was a different, un-defined variable until I realised it was a typo.
- You never actually define the [,] notation you use in the figures, which are the "sequence identity ranges". I'm glad that it is a different bracket type from the parameter sets you define (a,d), but it still needs to be defined. I only "got it" because you say you choose values from a 40-70% similarity range and I made the connection.
- Taking a look at this sentence, I think the second part is not accurate:
"Safety coverage is defined by the portion (%) of a protein sequence which is reported by EMERALD as (alignment-) safe, while stable coverage is the portion (%) of a sequence that is considered stable according to
the distinct protein-structural features classification (userdefined) described in the previous section."
The first part makes sense:
safety coverage = Emerald safe bases / total bases
The second part sounds like it should be:
stable coverage = bases defined as stable by the user / total bases

It that is the case, then its distribution should be independent of what parameters are used in EMERALD, especially since the same number of sequences are used in each distribution and their lengths are presumably not changing. However, there are different distributions in the supplementary materials! My guess is that the bases that are included in the numerator and demoninator are filtered to only include those that have been aligned up to delta-safety, but this is written nowhere in the text. Note that I am interpreting your results section with this change in mind.

-Maybe add a sentence about how it is easy to change the substitution matrix, so this could potentially be used for other sequence types, like DNA/non-coding RNA, as this will enhance to applicability of your approach.

**Reviewer 2**

**Were you able to assess all statistics in the manuscript, including the appropriateness of statistical tests used?**

**Yes:** They use some simple comparisons between distributions and some simple metrics typical for machine learning performance evaluation

**Were you able to directly test the methods?**

No

**Comments to author:**

You present a method to extract sub-optimal alignments in a pairwise alignment between two (divergent) protein sequences and then use that suboptimal alignment information to infer a 'safety' for the alignment in regions thereof. The alignment-safe regions tend to correspond with secondary structure elements within the proteins. Whilst I appreciate the approach and its intent to use in essence the variability of the alignment to detect where it is reliable (and where not), I do question the way the method is tested, the description of some of the processes you use, and finally the applicability of the method.

* Major comments:

1. A major problem in sequence alignment is the definition of what constitutes the 'ground truth' - i.e. for proteins the amino acids that relate back to the same original position in the ancestral protein. In your approach, you use the definition of 'stable' residues, all the ones that are have STRIDE classifications that are not 'Coil', 'Strand' or 'Turn'. You then argue that the alignments that are 'safe' according to your method also tend to be 'stable' and use that as a validation of your approach, where you define true positives as stable/safe positions, and so forth. However, this completely ignores a couple of underlying issues. The first is the influence of amino acid composition in relation to stable secondary structure elements. It is well known that certain amino acid substitutions in alpha helices or beta sheets are well tolerated by evolution. These will then also give higher scores in BLOSUM62 type matrices, which will, in my understanding of your approach, also result in more/better suboptimal alignments and a generally 'safer' alignment in those regions. You should therefore carefully discuss and explore the influence of amino acid bias in the 'stable' regions on your method performance. Second, your approach does not take into account if the 'safe' alignments are in fact also correct, only that they tend to correspond to secondary structure element regions. This concept (secondary structure elements align better) has been known for a long time and has also been actively used in several alignment methods like t-COFFEE. A much more challenging (but much fairer) evaluation of your method would be to use structure-based alignments between divergent sequences (based for example on AlphaFold2, see https://academic.oup.com/bioinformatics/article/38/22/5007/6709341), then check whether the regions that align well in the structure alignment (non gapped) are 'safe', and whether the regions that you consider 'safe' are in fact aligned well. In your case, this would probably mean that the correct structure-based alignment is in the sub-optimal solution space? At the moment, you could have alignments that are considered safe and stable but are completely incorrect, for example because they align two alpha helical regions that correspond to different helices. Third, alpha helices and beta sheets are also known to evolve at different rates, how does that impact your evaluation, and what about intrinsically disordered regions where you might have conserved elements such as motifs that might align well? Without addressing these points above, I do not see what your method contributes to the field.

2. In relation to the description of some of the processes, please consider the following points. i) You are using two sequences for the alignment, what if out of the two aligned residues one is 'stable' and one is not? Or do you not take that into account? ii) Although your datasets are available online, the method section does not describe how you generated them. There are mentions in the results, but the procedure to obtain them and the methods run on them should be more precisely defined in the methods section. For example, you mention using STRIDE, which works from structure coordinates - did you use PDB ones and/ore AlpahFold2 models? In general, you focus only on the math behind the approach, but not on the data and validation, this is not acceptable in this context.

3. Even if your method gives new insights, how can one use it in practice? Say there are 10 suboptimal alignments to choose from, which one is 'best'? What do I do with the parts that are 'not safe'? You should at least try to describe how one could use your method in practice.

* Minor comments:

- Your final sentence of the introduction implies that somehow your method would help to detect the effect of point mutations, which you relate to the AlphaFold2 failure to do so? This does not make sense - the reason AF2 does not detect the effect of point mutations well is because it relies on a multiple sequence alignment (MSA) of many sequences from which it detects pairwise amino acid connections - changing one residue will not drastically change its 'conclusions' from that MSA. You should clarify what you mean by this sentence, or remove it.

- Why is ensuring the 'biggest gap between medians' (l36-37, p5) the best trade-off between safety and stable structure retention?

- Why are MSAs or HMMs 'often impractical'? Many of these approaches are lightning fast and work very well, can you support your statement?

- You state that 'alignment-safe positions seem to encode the backbone of protein structure'. What does that mean? The protein always has a backbone, this is defined by its chemical bonding pattern, it is not 'encoded'. You might mean 'stably folded backbone' here, but some of the terminology you use (lik here) is confusing and seems to indicate a lack of certain basic biophysical concepts in relation to proteins.

- Please avoid statements like 'stimulate a renaissance of sequence alignment research' (Conclusions). This is unnecessary and highly unlikely - there has never been a lack of research and insights in this area, as it constitutes the 'bread and butter' of many bioinformatics methods.

- You might want to consider using 'reliable' instead of 'safe'.

**Authors Response**

**Point-by-point responses to the reviewers’ comments:**

Reviewer #1: Overall, I think that the motivation for the package is scientifically sound and interesting. I have a specific project in mind which would benefit from using this package.

Response: We would like to thank this reviewer for taking the time to carefully assess our methodology and application and for providing such constructive feedback which allowed us to significantly improve EMERALD and our manuscript.

Major changes:

- One thing that would be of interest and would demonstrate whether this method is valuable is the distribution of the lengths of the contiguous stable intervals. If the method ends up picking lots of very short robust matches it may be less useful than having longer contiguous intervals. I'm sure this is going to change a lot on the basis of the parameter choice too, so it would be good to help the reader see how to pick these parameters for various applications.

Response: This is an excellent point and we now address this aspect of the contiguous stable intervals by providing the summary statistics of these distributions as Supplementary Table 1. The intuition of Reviewer #1 was spot on. For example, we found that for all identities for alpha = 75% and Delta = 8, the average safety window length covers around 12.65% of the average sequence length. In the manuscript we clarify this contiguous stable interval aspect by writing “The choice of parameters also has an impact on the contiguity of safety window lengths and counts. For example, when selecting very conservative parameter settings, a larger proportion of shorter safety windows are generated. To buffer this effect, we added the additional constraint as a new EMERALD parameter –m (--windowmerge) which determines whether short safety windows that appear in close proximity (i.e., intersecting or adjacent to each other), are subsequently joined into one large safety window. We quantify this parameter selection behaviour in Supplementary Table 1, which now provides the average sequence and safety window lengths (summary statistic of contiguous stable intervals). As expected, lowering the pairwise sequence identity results in shorter and overall less contiguous safety windows. Additionally, a substantial amount of the safety windows of sequence alignments in the [70%,100%] identity range have a length exceeding 50% of their full sequence length.” in the Results section.

- While the code is available for the pipeline with DIAMOND, etc, as far as I can see, the benchmarking code isn't available/re-usable.

Response: We apologise for this confusion. While our benchmarking code was provided as Jupyter Notebooks via the Figshare link, most readers will intuitively consult GitHub when searching for computationally reproducible scripts (since we also provide the GitHub link as well). We now unify this and provide all computationally reproducible analysis scripts under https://github.com/algbio/emerald-analysis (now also including the benchmarking code).

- Since you are doing it anyways, why not report the proportion of stable sequence by stride assignment (EMERALD-safe bases in AlphaHelix/Total AlphaHelix bases, etc). Boom, now you have a really interesting new scientific discovery with your tool to showcase an application of your method. This is the obvious scientific question raised by your method and it would save people a lot of effort/time to just report these results, since this is already calculated!

Response: We are very grateful to Reviewer #1 for this fantastic suggestion. We now report the proportion of stable sequence by stride assignment and show the results in Suppl Figure 2. In the manuscript, we now write: “In addition to Figure 2, we also assessed the safety coverage of each individual amino acid structure type in Supplementary Figure 2, which shows that unstable amino acids have a smaller safety coverage than stable ones. Coil has the smallest coverage followed by Turn, with an exception of Strand having a higher coverage than AlphaHelix, which we defined as stable. EMERALD sufficiently captures amino acids of type Bridge and 310Helix, despite STRIDE only assigning these types to 0.8% and 3% of all amino acids, respectively.”. The intuition of Reviewer #1 was indeed correct.

- You clearly compare against the obvious control (delta=1). However, I think it may also be important to compare against a multiple sequence alignment, as comparing multiple sequences at the same time may help "bridge the gap" between poorly aligned/divergent sequences.

Response: This is an excellent discussion point. While overall consensus in the alignment community agrees that MSAs tend to be more sensitive than pairwise alignments when aligning fairly similar sequences, in the MSA construction step, pairwise optimal alignments are conceptually “joined” into a MSA. In other words, the optimal alignment bias is magnified in the MSA construction. This in fact is one of the main motivations behind EMERALD, because it can perform fairly well when dealing with highly divergent sequence clusters and when scaling to the protein biodiversity of the tree of life. Instead of focusing on placing highly divergent sequences into one MSA, we specialise on revealing the few alignment-safe windows that are part of the true (unobserved) alignment, in the sense that they are the same across all optimal and suboptimal alignment solutions (Privman, 2012; Penn, 2010; Ashkenazy, 2014).

We also address this point in a response to Reviewer #2, where we added the following new statement: “The concept of solution-safety (Tomescu and Medvedev, 2017) guarantees the following property. If the true alignment-configuration is inside the full solution space of the dynamic programming matrix (i.e., optimal and ∆-suboptimal alignment-configurations), then windows that are the same across all such alignment-configurations (safety windows) are also part of the true alignment (which cannot be observed in reality). In other words, analogous to sampling theory in statistics where the ground truth of a data universe cannot be measured, we assume that a representative sample drawn from this data universe allows us to infer rules and principles about the data universe itself. Alignment-safety windows can thus be seen as analogy of a representative sample drawn from the true (unobserved) alignment. The biological relevance of this approach can then be studied by annotating these alignment-safe windows/positions in regard to their overlap with protein secondary structural features, their contribution to protein selection pressures such as synonymous vs nonsynonymous substitution rates (dN/dS), and mappings onto AlphaFold2 structures or their underlying multiple-sequence alignments (MSAs).”.

In response to an analogous request of Reviewer #2, we revealed that we are currently preparing a manuscript where we systematically study the different algorithms of MSA construction and the conserved sequence blocks they can reveal compared to the safety-windows generated with EMERALD in the context of a comprehensive biological study. Since we submitted this manuscript as a Method article, we did not want to mix our methodological description and agnostic applicability of EMERALD with a particular biological study.

Easy fixes, but extremely important changes:
- You need to include x- and y- axis labels on all figures! It is very annoying to have to read the legend to figure this out.

Response: We apologise for this inconsistency and now added sufficient x- and y-axis labels to all figures.


- You have to capitalise ALL your deltas. I thought it was a different, un-defined variable until I realised it was a typo.

Response: We also apologise for this inconsistency and now fixed the typo.

- You never actually define the [,] notation you use in the figures, which are the "sequence identity ranges". I'm glad that it is a different bracket type from the parameter sets you define (a,d), but it still needs to be defined. I only "got it" because you say you choose values from a 40-70% similarity range and I made the connection.

Response: We now added a clarification of the [,] notation for the sequence identity ranges and write in the Results section (under “Benchmarking and analysis of stable versus unstable protein structural features that are encoded by alignment-safe windows”): “We represent the sequence identity ranges as intervals of real numbers with notation [i,j] where i defines the lower identity boundary and j the upper identity boundary.”.

- Taking a look at this sentence, I think the second part is not accurate:
"Safety coverage is defined by the portion (%) of a protein sequence which is reported by EMERALD as (alignment-) safe, while stable coverage is the portion (%) of a sequence that is considered stable according to
the distinct protein-structural features classification (userdefined) described in the previous section."
The first part makes sense:
safety coverage = Emerald safe bases / total bases
The second part sounds like it should be:
stable coverage = bases defined as stable by the user / total bases

It that is the case, then its distribution should be independent of what parameters are used in EMERALD, especially since the same number of sequences are used in each distribution and their lengths are presumably not changing. However, there are different distributions in the supplementary materials! My guess is that the bases that are included in the numerator and demoninator are filtered to only include those that have been aligned up to delta-safety, but this is written nowhere in the text. Note that I am interpreting your results section with this change in mind.

Response: We apologise for the confusion, indeed the distribution of the stable coverage stays the same for all EMERALD parameters. In Supplementary Figures 2-4, the number of sequences should stay constant, which we have fixed now. Previously, we mistakenly removed sequences with zero safety coverage, since we also do this for calculating the precision in order to avoid dividing by zero. After the fix, no differences were observed in the new plots.

-Maybe add a sentence about how it is easy to change the substitution matrix, so this could potentially be used for other sequence types, like DNA/non-coding RNA, as this will enhance the applicability of your approach.

Response: This is an excellent suggestion. We now incorporated this by stating “This makes EMERALD extendable to sequence alignments beyond protein sequences.” in the Methods section (under “Implementing Alignment-Safety into the scientific software EMERALD”).

Reviewer #2: You present a method to extract sub-optimal alignments in a pairwise alignment between two (divergent) protein sequences and then use that suboptimal alignment information to infer a 'safety' for the alignment in regions thereof. The alignment-safe regions tend to correspond with secondary structure elements within the proteins. Whilst I appreciate the approach and its intent to use in essence the variability of the alignment to detect where it is reliable (and where not), I do question the way the method is tested, the description of some of the processes you use, and finally the applicability of the method.

Response: We are very grateful to Reviewer #2 for taking the time to prepare constructive feedback that allowed us to identify clear weaknesses of our communication and for suggesting excellent improvements to the EMERALD methodology and its possible biological applications.

* Major comments:

1. A major problem in sequence alignment is the definition of what constitutes the 'ground truth' - i.e. for proteins the amino acids that relate back to the same original position in the ancestral protein. In your approach, you use the definition of 'stable' residues, all the ones that have STRIDE classifications that are not 'Coil', 'Strand' or 'Turn'. You then argue that the alignments that are 'safe' according to your method also tend to be 'stable' and use that as a validation of your approach, where you define true positives as stable/safe positions, and so forth. However, this completely ignores a couple of underlying issues. The first is the influence of amino acid composition in relation to stable secondary structure elements. It is well known that certain amino acid substitutions in alpha helices or beta sheets are well tolerated by evolution. These will then also give higher scores in BLOSUM62 type matrices, which will, in my understanding of your approach,
also result in more/better suboptimal alignments and a generally 'safer' alignment in those regions. You should therefore carefully discuss and explore the influence of amino acid bias in the 'stable' regions on your method performance. Second, your approach does not take into account if the 'safe' alignments are in fact also correct, only that they tend to correspond to secondary structure element regions. This concept (secondary structure elements align better) has been known for a long time and has also been actively used in several alignment methods like t-COFFEE.

Response: Thank you for this excellent discussion and for the opportunity to incorporate a more sophisticated discussion on the amino acid bias induced by substitution matrices in our revised manuscript. We apologise for the miscommunication since our approach does not define 'stable' residues as all the ones that have STRIDE classifications and are not 'Coil', 'Strand' or 'Turn'. In fact, the context of ‘residue safety’ refers to the fact that our algorithmic method attempts to agnostically reveal the amino acid positions within a pairwise alignment that are the same (constant) across all possible suboptimal alignment configurations within the threshold-boundaries of the suboptimal alignment space. In other words, alignment-safety refers to the property that ‘safe positions’ remain the same in all suboptimal alignment configurations and are thus ‘safe from alternative alignment placings’ (for this reason we named this method ‘alignment-safety’). We leave it then up to users to explore which substitution matrix and ‘ground truth’ dataset best represents their use-case. We then used the STRIDE annotation purely as an example for running EMERALD and not as a result or biological claim. We simply meant to illustrate how users can biologically benchmark/test/validate our algorithmically determined alignment-safe positions when they retrieve them after running our software.

We hope that this point clarifies why we wrote in the manuscript: “While this classification is naive in the first instance (for example, because there are well-known cases where Coils, Strands and Turns can be stable to fix certain protein conformations and vice-versa, alpha-helices can be fairly flexible and unstable (Levy Karin et al. [2019], Bondos et al. [2021])), we use this distinction only to exemplify how users can categorize known protein-structural features into distinctive structural feature classes to benchmark the suboptimal alignment-space for their domain-specific application and quantification of biological relevance. The main motivation behind such categorization is to test which regions of a protein structure are robustly encoded in alignment-safe intervals. We envision that users make extensive use of this benchmarking setup to explore, quantify and test the biological relevance of harnessing the suboptimal alignment-space for encoding structural features and protein evolution of their (divergent) sequences of interest.” (Results under “Defining Alignment-Safety for Pairwise Protein Sequence Alignments”).

We realised that this misunderstanding may stem from our sentence “In order to assess biologically relevant outcomes from quantifying alignment-safety, we assume that the ability to retain structural information known to influence protein folding such as alpha-helices, loops, etc while compressing the full primary protein sequence to alignment-safe windows is the ‘ground truth’ of biological relevance when comparing fold structures using primary sequences alone”.

We now rewrote this sentence to make it more clear that we don’t assume that the STRIDE annotation is our ‘ground-truth’, but rather that since our software is agnostic we don’t know the ‘ground-truth’ of a particular user-application and therefore encourage users to annotate the output of EMERALD against a plethora of protein features.

We now write: “In order to annotate which biological features alignment-safe residues may encode in a particular threshold- and substitution-matrix configuration, we use the STRIDE annotation such as alpha-helices and loops to exemplify how users can biologically assess the output of EMERALD. However, we would like to point out that users need to pay close attention to what suffices as valid ‘ground truth’ for their benchmarking, since EMERALD is agnostic to particular biological applications and the choice of substitution matrix may induce a well characterized amino acid bias for evolutionary more stable residues (Notredame et al., 2000; Chatzou et al., 2016). Recent advancements in protein structure prediction can also provide users with a more structurally oriented validation dataset (Baltzis et al., 2022).”

Reviewer #2 makes two excellent points which we failed to discuss in detail and now incorporate in the revised version of the manuscript:

“You should therefore carefully discuss and explore the influence of amino acid bias in the 'stable' regions on your method performance.”

We completely agree with this assessment, because users may not be fully aware of the systematic bias any substitution matrix can induce when benchmarking against a certain ‘ground truth’ (which we agree is a major problem in validating sequence alignments). We therefore added a more concrete discussion in the section above (illustrated in red). Overall, our violin plots show that in parameter settings close to optimal alignments (e.g. delta = 0, identity range 40-70%) safety windows cover almost the entire query/subject sequence and are thus not more meaningful (or biased) than classic pairwise alignments. Only when going into the suboptimal alignment space safety windows become biologically revealing. Reviewer #1 also correctly observed that a fair control for our agnostic approach could be delta = 1, which we addressed as suggested.

“Second, your approach does not take into account if the 'safe' alignments are in fact also correct, only that they tend to correspond to secondary structure element regions. This concept (secondary structure elements align better) has been known for a long time and has also been actively used in several alignment methods like t-COFFEE.”

Thank you for pointing out this misconception. As stated above, we did not intend to claim any biological novelty and we completely agree that the concept that secondary structure elements align better is well known (especially under the regime of a BLOSUM62 matrix). To clarify this point we now also add the following statement:
“While it is well understood that secondary structure elements align better than non-secondary structure elements or disordered parts of proteins, our methodology can reveal the concrete regions of a protein sequence (alignment-safety windows) that are the same across all (considered) suboptimal alignment solutions. This feature of EMERALD allows users to explore how robust certain sequence regions are to alternative alignment configurations and different substitution matrices.”.

A much more challenging (but much fairer) evaluation of your method would be to use structure-based alignments between divergent sequences (based for example on AlphaFold2, see https://academic.oup.com/bioinformatics/article/38/22/5007/6709341), then check whether the regions that align well in the structure alignment (non gapped) are 'safe', and whether the regions that you consider 'safe' are in fact aligned
well. In your case, this would probably mean that the correct structure-based alignment is in the sub-optimal solution space? At the moment, you could have alignments that are considered safe and stable but are completely incorrect, for example because they align two alpha helical regions that correspond to different helices. Third, alpha helices and beta sheets are also known to evolve at different rates, how does that impact your evaluation, and what about intrinsically disordered regions where you might have conserved elements such as motifs that might align well? Without addressing these points above, I do not see what your method contributes to the field.

Response: In the light of the miscommunication clarified in point a), we completely agree with Reviewer #2 that many suboptimal solutions can be “incorrect” when attempting to align two alpha helical regions that correspond to different helices. However, our method does not attempt to find one suboptimal solution that is superior to an optimal or another suboptimal solution, but rather has the purpose to identify the amino acid positions that are the same across ALL (optimal AND suboptimal) alignment configurations (Figure 1, green areas). In other words, our method reveals how many and which exact amino acid residues are robust to alternative alignment placings. The concept of solution-safety from which our alignment-safety approach is derived suggests that since alternative solutions in the (suboptimal) solution space are confounding without further constraints on this space, then safety windows are one tool allowing us to identify alignment regions that are mathematically guaranteed to be part of the "true" solution (since we assume the true solution is somewhere within the solution space). In other words, the core assumption behind safety-windows is that while we can never see the “true best alignment”, if we assume that this “true best alignment” lies somewhere within the solution space, safe-positions are a subset of the “true best alignment”, since they are shared among all alternative alignment configurations in this solution space. The next step then is to annotate which biological features (e.g. protein structural features) these safe positions encode (see also response to a few points below).

Reviewer #2 correctly recognised the impact that such a method has when exploring alignment-safe positions in a broad context of protein biology such as AlphaFold structural mapping, positional constraints on protein evolution (dN/dS), co-evolving loci, protein-protein interactions etc. Since we submitted this manuscript as a Method article that will facilitate a broad range of original research, we did not intend to mix both Method and biological study in one manuscript at this stage. In fact, we are already preparing another manuscript where we employ EMERALD in a large-scale scientific study where original results and biological relevance are presented in the context of a concrete biological question which incorporates sequence constraints (dN/dS) and mapping onto AlphaFold2 structures.

2. In relation to the description of some of the processes, please consider the following points.

i) You are using two sequences for the alignment, what if out of the two aligned residues one is 'stable' and one is not? Or do you not take that into account?

Response: We only consider residues as alignment-safe (alternative phrasing would be “robust to different alignment configurations”) when they are the same amino acid or same alignment pair across ALL optimal AND suboptimal alignment configurations (Figure 1, green areas). For example, when one alignment configuration at sequence position one has a particular amino acid and another suboptimal alignment configuration has a different amino acid at sequence position one, we would not consider sequence position one as alignment-safe (we prefer not to use the term ‘stable’, because this term may get confused when attempting to map this sequence position onto the corresponding protein structural position, e.g. secondary structure). If ‘stable’ in the reviewer comment refers to the STRIDE annotation, please note that our safety computation does not use any STRIDE information, it is a purely algorithmic (agnostic) process exploring (what is common / safe in) the space of optimal and suboptimal alignment solutions.

ii) Although your datasets are available online, the method section does not describe how you generated them. There are mentions in the results, but the procedure to obtain them and the methods run on them should be more precisely defined in the methods section. For example, you mention using STRIDE, which works from structure coordinates - did you use PDB ones and/or AlphaFold2 models? In general, you focus only on the math behind the approach, but not on the data and validation, this is not acceptable in this context.

Response: We appreciate the feedback that our methodological description would benefit from more details (see also request made by Reviewer #1). We would like to point out that in the submitted version of the manuscript we wrote in (Results, In silico
experimental design to investigate the biological relevance of alternative suboptimal alignment configurations): “[...] To achieve this, we retrieved the Swiss-Prot database from [Bairoch and Apweiler, 1997] (June 2021), which represents a manually curated subset of the UniProtKB [Consortium, 2021] database. In the version of June 2021, Swiss-Prot contains approximately 560k protein sequences which can be retrieved as a FASTA-file. After data retrieval, we filter out protein sequences that did not have corresponding AlphaFold2 predicted three-dimensional structures [Jumper
et al., 2021, Varadi et al., 2022]. We then clustered this dataset with DIAMOND DeepClust, a new sensitive deep-sequence-clustering method implemented into DIAMOND since version 2.1.0 [Buchfink et al., 2021]. We filter out cluster members with no stable bases and clusters of size 1, resulting in 15934 clusters and 396k sequences in total. To achieve a comprehensive overview of deep-homology associations between proteins across the full diversity intrinsic to the 396k Swiss-Prot sequences, clustering was carried out using a percent-identity threshold of 20% and 75% length coverage. [...]”.

In addition, we provided detailed analysis scripts which intend to illustrate how users can fully reproduce our data retrieval and analysis steps (listed under section “Data Access”): https://github.com/algbio/emerald-analysis. As was noted by Reviewer #1, while we initially provided all computationally reproducible scripts under the Figshare link, we did not communicate this well enough in our manuscript and GitHub repository. To solve this confusion, we now also incorporated the Figshare code into our GitHub repository https://github.com/algbio/emerald-analysis.

3. Even if your method gives new insights, how can one use it in practice? Say there are 10 suboptimal alignments to choose from, which one is 'best'? What do I do with the parts that are 'not safe'? You should at least try to describe how one could use your method in practice.

Response: We hope our discussion above clarified that our approach does not choose one particular suboptimal alignment configuration to claim “biological superiority” over the score-derived optimal alignment, but rather attempts to reveal the positions within a pairwise alignment that are part of the “true (but never fully observable) alignment” under the assumption of solution-safety (Tomescu and Medvedev, 2017; doi:10.1089/cmb.2016.014), where positions that are robust across ALL alignment configurations are marked as alignment-safe. In the example of Figure 1, we have 7 suboptimal and 1 optimal alignments, and three safety windows shared across an alpha proportion of these alignments (for alpha=75%). To further clarify this point we now added the following statements to the manuscript (section In silico experimental design to investigate the biological relevance of alternative suboptimal alignment configurations): “The concept of solution-safety (Tomescu and Medvedev, 2017) guarantees the following property. If the true alignment-configuration is inside the full solution space of the dynamic programming matrix (i.e., optimal and ∆-suboptimal alignment-configurations), then windows that are the same across all such alignment-configurations (safety windows) are also part of the true alignment (which cannot be observed in reality). In other words, analogous to sampling theory in statistics where the ground truth of a data universe cannot be measured, we assume that a representative sample drawn from this data universe allows us to infer rules and principles about the data universe itself. Alignment-safety windows can thus be seen as analogy of a representative sample drawn from the true (unobserved) alignment. The biological relevance of this approach can then be studied by annotating these alignment-safe windows/positions in regard to their overlap with protein secondary structural features, their contribution to protein selection pressures such as synonymous vs nonsynonymous substitution rates (dN/dS), and mappings onto AlphaFold2 structures or their underlying multiple-sequence alignments (MSAs).”.

* Minor comments:

- Your final sentence of the introduction implies that somehow your method would help to detect the effect of point mutations, which you relate to the AlphaFold2 failure to do so? This does not make sense - the reason AF2 does not detect the effect of point mutations well is because it relies on a multiple sequence alignment (MSA) of many sequences from which it detects pairwise amino acid connections - changing one residue will not drastically change its 'conclusions' from that MSA. You should clarify what you mean by this sentence, or remove it.

Response: We agree that this point is more confusing than useful and we removed it from the manuscript.

- Why is ensuring the 'biggest gap between medians' (l36-37, p5) the best trade-off between safety and stable structure retention?

Response: We agree that we should have been more precise in our explanation. The gap between the medians indicates that the stable structure retention is larger than the safety coverage throughout most sequences. This shows that EMERALD indeed captures secondary structural information (when annotated with STRIDE): If all residues were to be safe, the stable structure retention will be 100%, without revealing any further biological information. If all residues were chosen as safe with a probability of 50% uniformly (which is essentially throwing a coin for every amino acid) both the safety coverage and stable structure retention converge towards 50%. We attached the following sentences for clarification: “Similarly, if all amino acids are alignment-safe, the stable structure retention is equal to 100% meaning that no further biological information can be obtained. Thus, we are usually interested in an increased gap between the stable structure retention and the safety coverage.”

- Why are MSAs or HMMs 'often impractical'? Many of these approaches are lightning fast and work very well, can you support your statement?

Response: We agree that this phrasing is highly misleading and we did not mean to claim that MSAs or HMMs are often impractical in general (far from it). To eliminate this misunderstanding, we now removed this sentence from the text.

- You state that 'alignment-safe positions seem to encode the backbone of protein structure'. What does that mean? The protein always has a backbone, this is defined by its chemical bonding pattern, it is not 'encoded'. You might mean 'stably folded backbone' here, but some of the terminology you use (like here) is confusing and seems to indicate a lack of certain basic biophysical concepts in relation to proteins.

Response: We apologise for this unfortunate use of notation and agree that ‘stably folded backbone’ would have been the correct choice of phrasing. We now rewrote this sentence for clarification: “We interpret this result such that alignment-safe positions can be associated with the stably folded backbone of a protein structure and that further research is required to unveil the causal associations between alignment-safe positions and positions giving predictive signatures when inferring structural conservation above the twilight zone of protein evolution.”. We hope that overall our alignment-safety approach will stimulate further discussions in this direction within the alignment and comparative genomics fields.

- Please avoid statements like 'stimulate a renaissance of sequence alignment research' (Conclusions). This is unnecessary and highly unlikely - there has never been a lack of research and insights in this area, as it constitutes the 'bread and butter' of many bioinformatics methods.

Response: We agree and now removed this statement.

- You might want to consider using 'reliable' instead of 'safe'.

Response: While we agree that in a biological context the term ‘reliable’ would be more clarifying, the concept of alignment-safety is derived from the algorithmic concept of ‘solution safety’ (see Introduction where we refer to ‘solution safety’ and the reference Alexandru I. Tomescu and Paul Medvedev. Safe and complete contig assembly through omnitigs. J. Comput. Biol., 24(6):590–602, 2017. doi: 10.1089/cmb.2016.0141.). To maintain conceptual consistency, we prefer to use ‘safe’.

**Second round of review**

**Reviewer 1**

The authors have carefully addressed all my prior concerns.

**Reviewer 2**

Thank you for your extensive reply. I indeed misunderstood the core of your method, which is better and more extensively explained by the text you added. Identifying which regions of the alignment are 'safe' is indeed crucial, and your method does this elegantly by exploring the alignment space.

I have one remaining question however; there are many proteins that contain multiple domains that have very similar sequence composition. This would result in very good local alignments between these domains in the alignment space - could this confuse the detection of alignment-safety for such domains? If so, under which parameter conditions would this be more likely to happen?

**Authors Response**

**Point-by-point responses to the reviewers’ comments:**

Reviewer #2: Thank you for your extensive reply. I indeed misunderstood the core of your method, which is better and more extensively explained by the text you added. Identifying which regions of the alignment are 'safe' is indeed crucial, and your method does this elegantly by exploring the alignment space.

Response: Thank you very much for this very kind assessment.

I have one remaining question however; there are many proteins that contain multiple domains that have very similar sequence composition. This would result in very good local alignments between these domains in the alignment space - could this confuse the detection of alignment-safety for such domains? If so, under which parameter conditions would this be more likely to happen?

Response: This is an excellent point. Given that the domains are similar, we expect the suboptimal space to be narrow across them, because similarity in their composition will yield a high alignment score. As a result, such cases can indeed be captured by alignment-safety. For sequence pairs of high sequence identity (70%-100%), however, we envision results with very high safety coverage throughout the entire sequence space, while domains are captured best at lower identity levels. If we assume more divergent sequence compositions outside of the domain boundaries, we would recommend to choose a large parameter of at least 0.75, and to choose a parameter setting which is not too small (e.g. at least 6-8), since suboptimal alignments are likely to be closer to the optimum for less similar subsequences. To further explore this question in a more systematic way, we recommend to screen for an appropriate parameter by employing a new idea we refer to as ‘persistent safety-windows’: Increasing the parameter from 0 until safety-windows vanish, while storing the safety information in every iteration. This approach will inform a user about the robustness of safety-windows, that is for how long do subsequences stay alignment-safe, as safety-windows will continuously start to shrink and split. We believe that this addition will help users to capture important protein features such as repetitiveness or domain boundaries more accurately. For this reason, we now also created a feature-request issue on our Github repository (https://github.com/algbio/emerald/issues/1) to make the community aware of this suggestion and we will implement this feature into the EMERALD pipeline.
